# The Collagen-Modifying Enzyme PLOD2 Is Induced and Required during L1-Mediated Colon Cancer Progression

**DOI:** 10.3390/ijms22073552

**Published:** 2021-03-29

**Authors:** Sanith Cheriyamundath, Anmol Kumar, Nancy Gavert, Thomas Brabletz, Avri Ben-Ze’ev

**Affiliations:** 1 Department of Molecular Cell Biology, Weizmann Institute of Science, Rehovot 7610001, Israel; sanith.cheriyamundath@weizmann.ac.il (S.C.); anmol.kumar@atmiyauni.ac.in (A.K.); nancy.gavert@weizmann.ac.il (N.G.); 2 Experimental Medicine I, Nikolaus-Feibiger-Center for Molecular Medicine, University of Erlangen-Nuernberg, 91054 Erlangen, Germany; thomas.brabletz@fau.de

**Keywords:** PLOD2, L1, colorectal cancer, ezrin, invasion and metastasis, lysyl hydroxylase 2, colonic crypts

## Abstract

The overactivation of Wnt/β-catenin signaling is a hallmark of colorectal cancer (CRC) development. We identified the cell adhesion molecule L1CAM (L1) as a target of β-catenin-TCF transactivation in CRC cells. The overexpression of L1 in CRC cells confers enhanced proliferation, motility, tumorigenesis and liver metastasis, and L1 is exclusively localized in the invasive areas of human CRC tissue. A number of genes are induced after L1 transfection into CRC cells by a mechanism involving the cytoskeletal protein ezrin and the NF-κB pathway. When studying the changes in gene expression in CRC cells overexpressing L1 in which ezrin levels were suppressed by shRNA to ezrin, we discovered the collagen-modifying enzyme lysyl hydroxylase 2 (PLOD2) among these genes. We found that increased PLOD2 expression was required for the cellular processes conferred by L1, including enhanced proliferation, motility, tumorigenesis and liver metastasis, since the suppression of endogenous PLOD2 expression, or its enzymatic activity, blocked the enhanced tumorigenic properties conferred by L1. The mechanism involved in increased PLOD2 expression by L1 involves ezrin signaling and PLOD2 that affect the SMAD2/3 pathway. We found that PLOD2 is localized in the colonic crypts in the stem cell compartment of the normal mucosa and is found at increased levels in invasive areas of the tumor and, in some cases, throughout the tumor tissue. The therapeutic strategies to target PLOD2 expression might provide a useful approach for CRC treatment.

## 1. Introduction

Colorectal cancer (CRC) initiation and progression are characterized by the hyperactivation of the Wnt/β-catenin signaling pathway [1]. We identified the cell adhesion molecules L1 and Nr-CAM, members of the immunoglobulin superfamily of adhesion receptor proteins, as target genes of β-catenin-TCF signaling [2,3] and detected the co-expression of L1 with β-catenin at the invasive edge of human CRC tissue [3]. Overexpression of L1 in CRC cell lines confers enhanced motility, tumorigenesis and liver metastasis [4]. The analysis of genes induced by L1 expression in CRC cells revealed a number of genes known as intestinal stem cell signature genes [5,6,7,8,9]. We identified also additional L1-induced genes [10,11,12] that were all shown to be necessary for promoting the tumorigenesis and metastasis of CRC cells by L1 expression. A common signaling pathway activating these genes by L1 overexpression involves the cytoskeletal actin-binding protein ezrin and the NF-κB pathway [7,13]. The actin-binding cytoskeletal protein ezrin, preferentially localized in filopodia, is recruited after L1 transfection to the cytoplasmic tail of L1 at cell–cell contact sites [7,13]. After IκB binds to this complex, NF-κB is released from binding to IκB and migrates to the nucleus to activate target genes [7,13]. While analyzing genes that were induced by L1 whose levels were reduced by suppressing ezrin expression, we found that the level of lysyl hydroxylases 2 [encoded by the lysyl hydroxylase 2 (PLOD2) gene] was reduced in shEzrin-expressing CRC cells (Supplementary Table S1) [7]. PLOD2 is known to be an enzyme mediating the formation of stabilized collagen cross-links by the hydroxylation of lysyl residues in collagen [14]. The PLOD2-mediated changes in collagen organization are believed to play a key role in the migration and invasion of a wide variety of cancer cells [15]. In this study, we investigated the roles of PLOD2 in the L1-mediated promotion of CRC cell motility, tumorigenesis and liver metastasis.

## 2. Results

### 2.1. PLOD2 Levels Are Induced in L1-Overexpressing CRC Cells and the Expression of PLOD2 Is Suppressed by shEzrin

**Figure 1:**
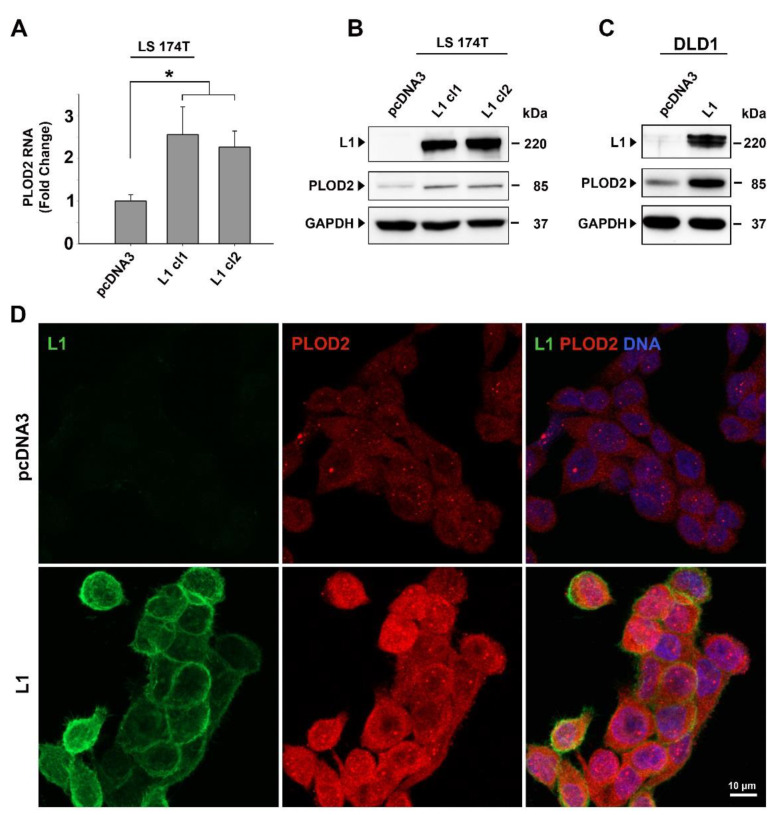
Induction of lysyl hydroxylase 2 (PLOD2) expression in colorectal cancer (CRC) cells by the overexpression of L1. (**A**) The level of PLOD2 RNA was determined in control clones of LS 174T transfected with pcDNA3 and in LS 174T CRC cell clones stably overexpressing L1 (L1 cl1 and cl2) by RT-PCR. (**B**) Western blot analysis of PLOD2 protein levels in the CRC cell lines described in (**A**). (**C**) Western blot analysis of PLOD2 protein levels in DLD1 CRC cells transfected with pcDNA3 or with L1. (**D**) Double immunofluorescence localization and levels of PLOD2 (red) and L1 (green) in LS 174T CRC cells expressing the control pcDNA3 plasmid or L1. DNA was visualized using DAPI (blue). * *p* < 0.05.

When analyzing the expression of genes whose levels were induced by L1 in CRC cell lines but reduced when ezrin levels were suppressed by shRNA to ezrin [7], we identified PLOD2 among these genes (Supplementary Table S1). The results of the RT-PCR experiments shown in Figure 1A demonstrate that the level of PLOD2 RNA was increased in CRC cell clones stably overexpressing L1 (Figure 1A, L1 cl1 and cl2), as compared to LS 174T cells expressing the empty pcDNA3 plasmid (Figure 1A, pcDNA3). Western blot analyses confirmed an increase in PLOD2 protein levels in LS 174T CRC cell clones overexpressing L1 (Figure 1B). The DLD1 CRC cell line overexpressing L1 also displayed an increase in PLOD2 levels (Figure 1C). This demonstrated that both of the CRC cell lines in which the Wnt/β-catenin pathway was activated by different mutations [β-catenin in LS 174T and adenomatous polyposis coli (APC) in DLD1] displayed an increase in PLOD2 upon L1 overexpression. CRC cells overexpressing L1 also showed an increase in endogenous PLOD2, as demonstrated by the immunofluorescence staining (Figure 1D). Taken together, these results suggest that increased L1 expression confers elevated endogenous PLOD2 expression in human CRC cell lines.

### 2.2. Modulation of PLOD2 Levels Affects the Proliferative and Motile Abilities of CRC Cells Expressing L1

**Figure 2:**
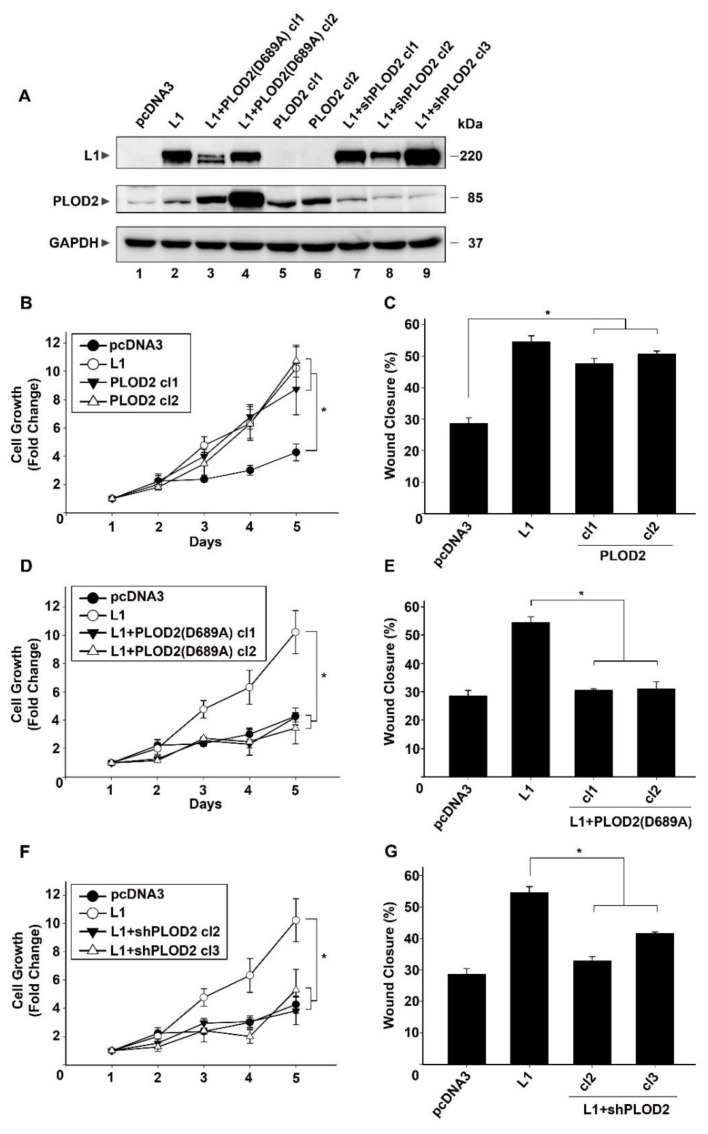
PLOD2 is required for the increase in cell proliferation and motility conferred by L1 in CRC cells. (**A**) The levels of endogenous and transfected PLOD2 proteins were determined in LS 174T cells stably expressing the control pcDNA3 plasmid (lane 1), L1 (lane 2), L1 plus a dominant negative PLOD2 mutant [L1 + PLOD2 (D689A) cl1 and cl2, lanes 3 and 4], PLOD2 (lanes 5 and 6) and L1 plus shRNA to PLOD2 (L1 + shPLOD2 cl1-cl3, lanes 7–9). (**B**,**D**,**F**) Analysis of the proliferation rates in serum-free medium of the cell clones described in (A) over 5 days. (**C**,**E**,**G**) Analysis of the motility of the cell clones described in (A) by the closure of “scratch wounds” introduced in the confluent monolayers of these CRC cell clones. * *p* < 0.05.

To examine the effects conferred by changes in PLOD2 levels on L1-mediated CRC cell proliferation and motility, we isolated individual human LS 174T CRC cell clones overexpressing PLOD2 (Figure 2A, lanes 5 and 6, compared to lane 1). We also isolated CRC cell clones overexpressing L1 in which the levels of PLOD2 were suppressed by shRNA to PLOD2 (Figure 2A, lanes 7–9 compared to lane 2). In addition, we prepared LS 174T cell clones overexpressing L1 in which we interfered with the enzymatic activity of endogenous PLOD2, by overexpressing a dominant negative (enzymatically inactive) PLOD2 (D689A) mutant [16] (Figure 2A, lanes 3 and 4). These different CRC cell clones displayed the expected increase, or decrease, in PLOD2 levels as compared to the control (pcDNA3)-expressing and L1-expressing CRC cell clones (Figure 2A, compare lanes 5 and 6 to lane 1, lanes 3 and 4 to lane 2 and lanes 7–9 to lane 2). 

These CRC cell clones were used to evaluate the changes conferred on cell proliferation (Figure 2B,D,F) and cell motility (Figure 2C,E,G) by modulating the PLOD2 levels in the L1-expressing and control LS 174T cells. As shown previously [3,4], the CRC cell clones transfected with L1 displayed a dramatically increased ability to proliferate in the absence of serum as compared to the control cells (Figure 2B, compare L1 to pcDNA3). This difference in proliferation was also observed when comparing the untransfected LS 174T cell line to L1-transfected LS 174T cells (Supplementary Figure S1). The overexpression of PLOD2 in the LS 174T cells was also very effective in stimulating the proliferation of these cells (Figure 2B, PLOD2 cl1 and cl2 compared to pcDNA3). The motility of these cells, as determined by the “scratch wound” closure method, was increased by PLOD2 overexpression, to an extent that was similar to that conferred by L1 overexpression (Figure 2C, PLOD2 cl1 and cl2 compared to L1 and pcDNA3). When the dominant negative PLOD2 (D689A) was expressed in the L1-transfected CRC cells, the effects conferred by L1 on CRC cell proliferation (Figure 2D, compare L1 to pcDNA3) and motility (Figure 2E) were reduced to the levels observed in the control LS 174T cells (Figure 2D, compare L1 to L1 + PLOD2 (D689A) cl1 and cl2). Similarly, when PLOD2 levels were suppressed by expression of a shRNA to PLOD2, the proliferation (Figure 2F, compare L1 to L1 + shPLOD2 cl2 and cl3) and motility (Figure 2G, compare L1 to L1 + shPLOD2 cl2 and cl3) of such CRC cells were reduced to the level observed in the control pcDNA3-transfected LS 174T cells. Taken together, these results suggest that the increased proliferative capacity in serum-free medium and in the motility of CRC cells conferred by L1 is dependent on the increase in PLOD2 levels. 

### 2.3. The L1-Mediated Increase in CRC Tumorigenesis and Liver Metastasis Require Elevated Expression of PLOD2

**Figure 3:**
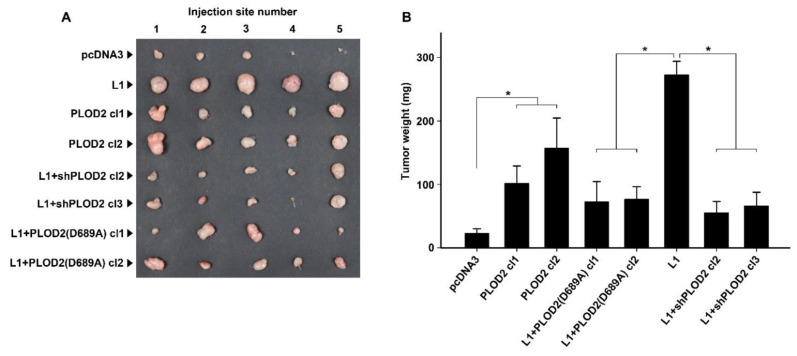
The L1-mediated increase in CRC cell proliferation in vivo requires PLOD2. (**A**) The cell clones described in Figure 2A were injected s.c. into immunocompromised nude mice and the tumors that developed were excised after eleven days. (**B**) Tumor weight was determined for the different CRC cell clones that were injected. Note the increase in tumor formation with L1-expressing (compare L1 to pcDNA3) and PLOD2-expressing cells (compare PLOD2 to pcDNA3) and the decrease in tumor development with cells expressing L1+ PLOD2 (D689A) and L1 + shPLOD2. * *p* < 0.05.

Next, we wished to determine the involvement of PLOD2 in the L1-mediated increase in CRC cell tumorigenesis and liver metastasis in vivo. Subcutaneous injection into nude mice of the CRC cell lines described in Figure 2A revealed that PLOD2 effectively stimulated tumor growth, albeit to a lesser extent than L1 (Figure 3A,B, compare PLOD2 cl1 and cl2 to L1). The suppression of endogenous PLOD2 activity in L1 overexpressing cells by the dominant negative PLOD2(D689A) mutant resulted in the suppression of the tumorigenic activity of L1-expressing CRC cells [Figure 3A,B, compare L1 + PLOD2(D689A) cl1 and cl2 to L1]. In addition, the reduction in endogenous PLOD2 levels in L1-expressing CRC cells, by the stable expression of shRNA to PLOD2, was also effective in blocking the increase in tumorigenic capacity conferred by L1 (Figure 3A,B, L1 + shPLOD2 cl2 and cl3, compare to L1).

**Figure 4:**
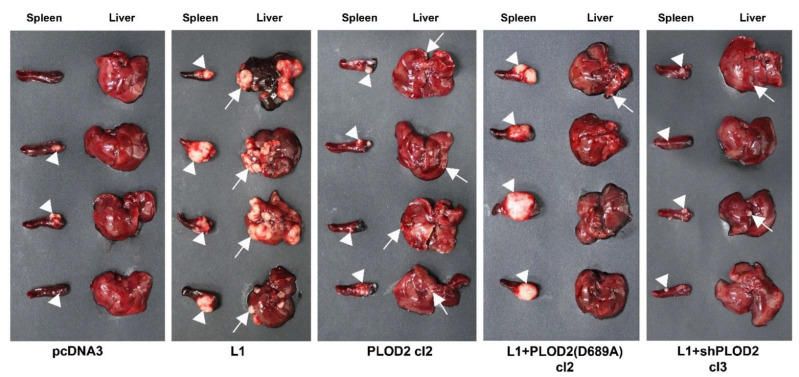
The liver metastasis of the CRC cells conferred by L1 transfection requires endogenous PLOD2 expression. The CRC cell clones described in Figure 2A were injected into the tip of the spleen in groups of four nude mice, and the development of tumors at the site of injection and liver metastasis were determined five weeks after the injection of the various CRC cell clones. White triangles mark tumor growth in the spleen. White arrows mark metastatic foci in the liver.

Since the human liver is the most common site of CRC cell metastasis, we wished to determine the possible role of PLOD2 in the increased liver metastasis of CRC cells conferred by L1 overexpression. The CRC cell clones described in Figure 2A were injected into the tip of the spleen in immunodeficient nude mice and the appearance of liver metastases was analyzed 5 weeks later. The results summarized in Figure 4 show that while L1 effectively promotes the liver metastasis of CRC cells, PLOD2 overexpression (on its own) in CRC cells is much less efficient in promoting metastasis (Figure 4, compare L1 to PLOD2 and to the control pcDNA3-expressing cells). The ability of L1 to confer liver metastasis in CRC cells was entirely dependent on PLOD2 expression, since the suppression of PLOD2 levels with shRNA to PLOD2 (Figure 4, L1 + shPLOD2) in L1-transfected cells, or the interference with PLOD2 enzymatic activity using the dominant-negative PLOD2 mutant [Figure 4, L1 + PLOD2 (D689A)], both blocked the metastatic capacity of L1-transfected CRC cells. As we have previously shown [4], tumor growth by CRC cells at the injection site in the spleen did not correlate with the metastatic capacity of these cells (Figure 4, Spleen). Taken together, these results demonstrate that the L1-mediated progress in tumorigenesis and metastasis requires PLOD2.

### 2.4. The Signaling by L1 via Ezrin That Induces PLOD2 Expression Involves SMAD2/3 

**Figure 5:**
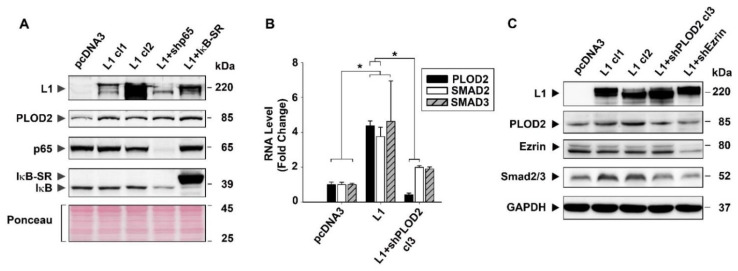
The induction in PLOD2 levels conferred by L1 transfection correlates with an increase in SMAD2/3 expression, but it is not affected by NF-κB signaling. (**A**) The levels of PLOD2 were determined in CRC cell clones expressing the control pcDNA3 plasmid, L1 (L1 cl1 and 2), L1 and an shRNA to the p65 NF-κB subunit (L1 + shp65) or L1 and a super-repressor of NF-κB signaling (L1 + IκB-SR). The levels of PLOD2, L1, p65 and IκB were determined by Western blotting with the relevant antibodies. Ponceau staining of the blots was used to determine equal protein loading. (**B**) RNA levels for PLOD2, SMAD2 and SMAD3 were determined by RT-PCR using the CRC cells stably expressing the control pcDNA3 plasmid, L1 and L1 + shRNA to PLOD2. (**C**) Western blot analysis of the levels of PLOD2, SMAD2/3 and ezrin in the CRC cell clones expressing the control pcDNA3 plasmid, L1 (L1 cl1 and cl2), L1 + shPLOD2 and L1 + shEzrin. GAPDH levels served to determine equal protein loading on the gel. Note the correlation between the changes in the levels of PLOD2 and SMAD2/3 in the different CRC cell clones. * *p* < 0.05.

In previous studies, we have shown that the increase in the expression of various genes in CRC cells following L1 transfection operates by a mechanism that involves cooperation between ezrin and NF-κB signaling [7,10,12,13]. Since the induction of PLOD2 by L1 depends on ezrin (Supplementary Table S1), we wished to determine the downstream effectors of the ezrin-mediated increase in PLOD2. We began by analyzing the levels of PLOD2 in L1-expressing CRC cells in which the signaling by NF-κB was compromised by the expression of a shRNA against the p65 subunit of NF-κB (Figure 5A, L1 + shp65), or by expressing a mutant form of IκB that suppresses the NF-κB pathway (Figure 5A, L1 + IκB-SR). The results presented in Figure 5A show that interference with the NF-κB pathway in L1-expressing cells did not significantly affect the levels of PLOD2 (Figure 5A, L1 cl1 and cl2, compared to L1 + shp65 and L1 + IκB-SR). This suggests that the induction of PLOD2 that depends on ezrin does not involve the NF-κB pathway. 

Since ezrin was also shown to collaborate with SMAD2/3 signaling in CRC cells [17], we determined by RT-PCR whether the levels of SMAD2/3 were affected by L1 overexpression in CRC cells. The results shown in Figure 5B demonstrate that L1 induced the expression of SMAD2/3 RNA (Figure 5B, L1 compared to pcDNA3) and that cells expressing L1 and shRNA to PLOD2 displayed a decreased level of both PLOD2 and SMAD2/3 RNA (Figure 5B, L1 + shPLOD2 cl3). These results on the level of RNA were also observed in PLOD2 and SMAD2/3 protein levels, as determined by Western blot analysis (Figure 5C). The increase (by L1) and decrease in PLOD2 expression (by shPLOD2 and shEzrin) was paralleled by a similar change in SMAD2/3 levels (Figure 5C, compare the SMAD2/3 levels to PLOD2 in L1-expressing and in L1 + shPLOD2 and L1 + shEzrin CRC cells). Together, these results suggest that the L1-mediated induction in PLOD2 requires ezrin, and since both shPLOD2 and shEzrin decreased SMAD2/3 levels, this implies that ezrin collaborates with PLOD2 to induce SMAD2/3. 

### 2.5. PLOD2 Is Localized in the Colonic Crypts Compartment of Normal Mucosa and at Increased Levels in Invasive Areas and throughout the Tumor Tissue

**Figure 6:**
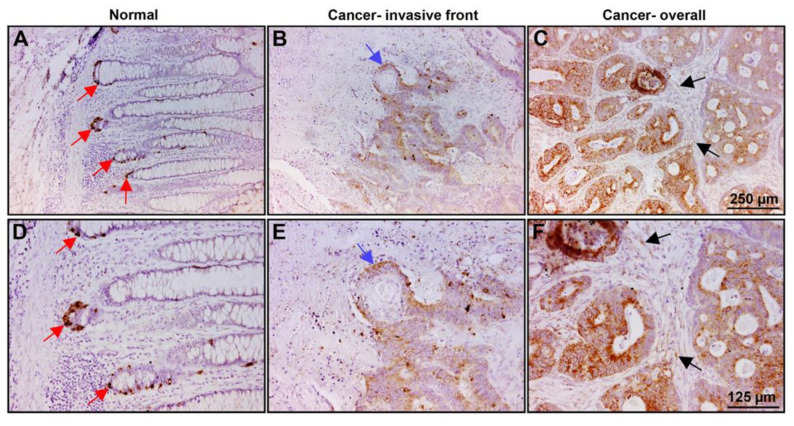
PLOD2 is localized in the stem cell compartment of the normal mucosa and at increased levels in the invasive front and throughout the CRC tissue. Samples of CRC tissue from 38 patients were paraffin-fixed and immunostained with an antibody against PLOD2. (**A**,**D**) PLOD2 is expressed in the stem cell compartment at the bottom of the crypts (red arrows) in the normal mucosa. (**B**,**E**) PLOD2 is localized at the invasive front of the CRC tissue (blue arrow). (**C**,**F**) In some tumors, PLOD2 is also expressed at increased levels throughout the tumor tissue, with a weak signal in the stromal tissue around the tumor (black arrows).

We wished to determine the localization and expression of PLOD2 in the normal mucosa and in human CRC tissue. Paraffin sections from 38 human CRC tissue samples were immunostained with an antibody against PLOD2 (Figure 6). The normal colonic mucosa was negative for PLOD2, except for the staining of the colonic crypts where the stem cell compartment is localized (Figure 6A,D, red arrows). Fifty percent (19/38) of tumor tissue samples showed positive staining for PLOD2, with 74% of the samples displaying preferential staining of cells at the invasive front of the tumor tissue (Figure 6B,E, blue arrow) and 26% stained throughout the tumor tissue (Figure 6C,F). In a few cases, weak staining for PLOD2 was also observed in the stroma around the tumor tissue (Figure 6C,F, black arrows). The results suggest that PLOD2 is localized in the colonic crypts in the stem cell compartment of normal mucosa. In the CRC tissue, PLOD2 is expressed at increased levels, with a preference for the invasive edge of the tumor, suggesting that PLOD2 is involved in promoting human CRC cell invasion.

## 3. Discussion

In this study, we identified PLOD2 among the genes that are induced, and which are required for the properties conferred by L1 overexpression in CRC cells during cancer progression. These include enhanced proliferation under stress, increased motility, tumorigenesis and liver metastasis. PLOD2 is a lysyl hydroxylase 2 enzyme that regulates the formation of stabilized cross-links in collagen [18]. The deposition of such modified collagen in the extracellular matrix (ECM) is believed to be a key facilitator of tumor cell motility, by forming a “highway” that enhances the invasion and metastasis of various types of cancer cells, including the tissues of the breast [19], liver [20], lung [21], pancreas [22], bladder [23], kidney [24], cervix [25], glia [26], sarcoma [16] and more [27]. In many of these cancers, clinical data have shown that the increased expression of PLOD2 is an effective and independent factor of poor prognosis and is associated with decreased survival [27]. In addition, some reported natural compounds that are suppressors of PLOD2 expression, such as minoxidil and berberine, were shown to inhibit cancer cell migration, reverse the switch in the formation of collagen cross-links and suppress metastasis in certain tumor types [16,28,29].

In previous studies, we have shown that L1 signaling that results in increased expression of several genes that are required for L1-mediated signaling in CRC cells involves an ezrin-NF-κB pathway [7,12,13]. However, we detected additional signaling pathways that were activated in response to L1 expression in CRC cells, including the β-catenin-TCF pathway [6], the STAT1 pathway [9] and the ILK pathway [8]. In the present study, we found that the induction of PLOD2 involves the SMAD2/3 pathway. It remains to be determined whether this pathway involves the direct activation of PLOD2 transcription via SMAD2/3 proteins. Previous studies in CRC cells have already demonstrated a collaboration of ezrin with SMAD2/3 signaling in the induction of PLOD2 gene transcription that involves the TGF-β receptor pathway, a key regulator of the epithelial to mesenchymal transition (EMT) process [17,27]. In this study, however, it appeared that ezrin and PLOD2 collaborated to induce SMAD2/3 levels.

Our analysis of human CRC tissue sections revealed that PLOD2 was exclusively expressed in the stem cell compartment (at the bottom of the colonic crypts) of the normal mucosa, while its expression was increased at the invasive edge of the CRC tissue and also throughout the tumor tissue (Figure 6). IGFBP2 was detected in a similar distribution in the CRC tissue and was also identified following ezrin suppression in the L1-expressing CRC cells ([7] and Supplementary Table S1). Interestingly, both genes were expressed in the stem cell compartment and were involved in the L1-mediated promotion of invasion and metastasis of CRC cells [30]. The immunohistochemical analysis of human CRC tissue also indicated that PLOD2 expression (in some cases) can be detected in the stromal compartment of the tumor (Figure 6C,F), supporting the important role of increased PLOD2 expression in the tumor microenvironment where collagen assembly plays a critical role in shaping the ECM for enhanced tumor cell migration/invasion. 

Since inhibitors of PLOD2 expression (minoxidil and berberine), and antifibrosis drugs, such as silibrin and pirfenodone, that regulate collagen organization, have already proven their efficacy in the treatment of various cancers [27], the expression of PLOD2 might prove to be a credible prognostic target in the treatment of the L1-mediated promotion of CRC invasion and metastasis. 

## 4. Materials and Methods

### 4.1. Cell Culture

The LS 174T cell line was grown in RPMI medium-1640 (Gibco, Thermo Fisher Scientific, Paisley, UK) containing 10% fetal bovine serum (FBS) (Gibco, Thermo Fisher Scientific, Paisley, UK) and 1% penicillin/streptomycin solution (Biological Industries, Israel). LS 174T+ pcDNA3 and LS 174T + L1 were maintained in RPMI medium 1640 containing neomycin (800 µg/mL). LS 174T + PLOD2 was maintained in RPMI medium 1640 containing puromycin (10 μg/mL). LS 174T-L1+ shPLOD2, LS 174T-L1+ PLOD2 (D689A), LS 174T-L1 + shp65 and LS 174T-L1 + IκB-SR were maintained in RPMI medium 1640 containing both neomycin (800 µg/mL) and puromycin (10 μg/mL). The LS 174T-L1 + shEzrin cells were maintained in RPMI medium 1640 containing both neomycin (800 μg/mL) and zeocin (500 μg/mL) [9,13].

### 4.2. Transfection, Cell Proliferation and Motility Assays

Stable transfection of LS 174T cells with PLOD2 and LS 174T-L1 with shPLOD2 or PLOD2 (D689A) was performed using the Xfect™ transfection reagent (Takara Bio, Mountain View, CA, USA) according to the manufacturer’s instructions. For cell proliferation assays, 10,000 cells were seeded in 12-well plates in the presence of medium containing 0.1% FBS, and the proliferation rate was determined by cell counting using a hemocytometer. Cell motility assays using the “scratch wound” method were performed as described [7].

### 4.3. Plasmids

PLOD2, shPLOD2 and PLOD2 (D689A) plasmids were a gift from T.S. Karin Eisinger-Mathason and M. Celeste Simon, Abramson Family Cancer Research Institute/Perelman School of Medicine, University of Pennsylvania [16].

### 4.4. Immunoblotting and Immunofluorescence

Immunofluorescence and immunoblotting were performed as described [13]. The antibodies used for immunoblotting were: rabbit anti-L1 (a gift from Dr. V. Lemmon, University of Miami, Miami, FL, USA) at 1:2000 dilution; mouse anti-PLOD2, GTX64917 (GeneTex, Hsinchu, Taiwan) diluted 1:1000; rabbit anti-phospho-IκBα, #2859 (Cell Signaling Technologies Inc., Danvers, MA, USA) diluted 1:1000; rabbit anti-NF-κB p65, sc-109 (Santa Cruz Biotechnology Inc., Dallas, TX, USA) diluted 1:1000; mouse anti-GAPDH, sc-47724 (Santa Cruz Biotechnology Inc., Dallas, TX, USA) diluted 1:2000; rabbit anti-ezrin/radixin/moesin, #3142 (Cell Signaling Technologies Inc., Danvers, MA, USA) diluted 1:2000; and rabbit anti-Smad2/3, #5678S (Cell Signaling Technologies Inc., Danvers, MA, USA). Cells were lysed in RIPA buffer containing a 1% protease inhibitor cocktail (Sigma, Merck, Kenilworth, NJ, USA). Western blots were developed using the enhanced chemiluminescence (ECL) method. Mouse anti-L1 sc-514360 (Santa Cruz Biotechnology, Inc., Dallas, TX, USA) was used for immunofluorescence, and both the L1 and PLOD2 primary antibodies were diluted 1:150. The secondary antibodies were Alexa Fluor 488-labeled goat anti-mouse IgG (Abcam, Cambridge, UK) diluted 1:1000 and Cy3-labeled goat anti-rabbit IgG (Jackson Immunoresearch Laboratories, West Grove, PA, USA) diluted 1:1000. Nuclei were stained using 4′-6-diamidino-2-phenylindole (DAPI, Sigma-Aldrich, St. Louis, MO, USA). Images were acquired using the Zeiss LSM 800 confocal microscope, equipped with the Zeiss objectives 40X/1.3 NA and the ZEN imaging software (Carl Zeiss Microscopy GmbH, Jena, Germany) and analyzed using the Image J and FIJI software.

### 4.5. Quantitative RT-PCR

Total RNA was isolated from cells using the Bio-Tri reagent (Bio-Lab, Jerusalem, Israel), according to the manufacturer’s protocol. First-strand cDNA was synthesized using SuperScript™ II reverse transcriptase (Invitrogen™, Thermo Fisher Scientific, Waltham, MA, USA) using random primers. Fast SYBR™ green master mix was used for the experiment (Applied Biosystems™, Thermo Fisher Scientific Inc., Vilnius, Lithuania). Details of the primers used are listed in Supplementary Table S2. Data analysis was performed using the ΔΔCT method with the StepOne software v2.3 (ThermoFisher Scientific, Waltham, MA, USA), where GAPDH was used as the endogenous control.

### 4.6. Tumor Growth and Metastasis Assays

Athymic nude-Foxn1^nu^ mice were used for these experiments. Subcutaneous tumor growth was induced as described [9,13]. Briefly, 1.5 × 10^6^ cells in 100 μL phosphate-buffered saline (PBS) were injected into different sites in the flanks of male nude mice and were sacrificed on day eleven after injection. The ability of cells to metastasize from the spleen to the liver was determined by injecting 1.5 × 10^6^ cells in 20 μL PBS into the distal tip of the spleen of 4–5-week-old male nude mice, as described in [13]. The animals were sacrificed after five weeks, and primary tumor formation in the spleen and metastasis appearance in the liver were examined.

### 4.7. Immunohistochemistry

Immunohistochemistry was carried out on 38 paraffin-embedded human colorectal adenocarcinomas using anti-PLOD2 polyclonal rabbit antiserum diluted 1:150, from antibodies-online.com, as previously described [13].

### 4.8. Statistical Analysis

Statistical significance was determined using the Student’s non-paired t-test. A *p* value of <0.05 was considered significant and marked by an asterisk in the Figures.

## Supplementary Materials

The following are available online at https://www.mdpi.com/article/10.3390/ijms22073552/s1, Table S1: Genes downregulated in LS 174T cells expressing L1 + shEzrin as compared to cells expressing L1, Table S2: Primers used for RT-PCR experiments. Figure S1: Proliferation of LS 174T, LS 174T-pcDNA and LS 174T-L1 cell clones in 0.1% serum.
