# Supplementary material for: The Collagen-Modifying Enzyme PLOD2 Is Induced and Required during L1-Mediated Colon Cancer Progression
Source: Int J Mol Sci. 2021 Mar 29;22(7):3552. doi: 10.3390/ijms22073552 (PMC8038063; doi:10.3390/ijms22073552)
Supplement: Supplementary file 1 [file ijms-22-03552-s001.pdf]

Supplementary Materials

The Collagen Modifying Enzyme PLOD2 is Induced and Required During L1-mediated Colon Cancer Progression

Sanith Cheriyamundath[1], Anmol Kumar[1], Nancy Gavert[1], Thomas Brabletz[2], and Avri Ben-Ze'ev[1,*]

[1] Department of Molecular Cell Biology, Weizmann Institute of Science, Rehovot, Israel; sanith.cheriyamundath@weizmann.ac.il; anmol.kumar@atmiyauni.ac.in; nancy.gavert@weizmann.ac.il; avri.ben-zeev@weizmann.ac.il

[2] Experimental Medicine I, Nikolaus-Feibiger-Center for Molecular Medicine, University of Erlangen-Nerenberg, Erlangen, Germany; thomas.brabletz@fau.de

* Correspondence: avri.ben-zeev@weizmann.ac.il

Table S1. Genes downregulated in LS 174T cells expressing L1+shEzrin as compared to cells expressing L1

Gene symbol	Gene description	Fold change
HOXA11	Homeobox A11	3.291
CST1	Cystatin SN	3.025
RHOBTB3	Rho-related BTB domain containing 3	2.673
LRP4	Low density lipoprotein receptor-related protein 4	2.622
MME	Membrane metalloendopeptidase	2.510
HMGCS2	3-hydroxy-3-methylglutaryl-coenzyme A synthase 2	2.493
SNORD5	Small nucleolar RNA, C/D box 5	2.293
KRTAP17-1	Keratin associated protein 17-1	2.274
PROX1	Prospero homeobox 1	2.201
HSPA1B	Heat shock 70 kDa protein 1B	2.191
SNORA13	Small nucleolar RNA, H/ACA box 13	2.190
ZNF487	Zinc finger protein 487	2.176
AIM1	Absent in melanoma 1	2.166
KRTAP9-4	Keratin associated protein 9-4	2.124
PLOD2	Procollagen-lysine, 2-oxoglutarate 5-dioxygenase 2	2.110
KIAA1324	Estrogen induced gene 121	2.029
HSPA1A	Heat shock 70 kDa protein 1A	2.016
IGFBP2	Insulin-like growth factor-binding protein 2	2.012

Supplementary Table 1. cDNA microarray analysis of genes whose expression is induced in L1-expressing CRC cells as compared to CRC cells expressing the pcDNA3 control plasmid and whose levels are suppressed in cells expressing L1 plus shRNA to ezrin.

Figure S1. Analysis of the proliferation rate in LS 174T, LS 174T+pcDNA3 and LS 174T+L1 cells

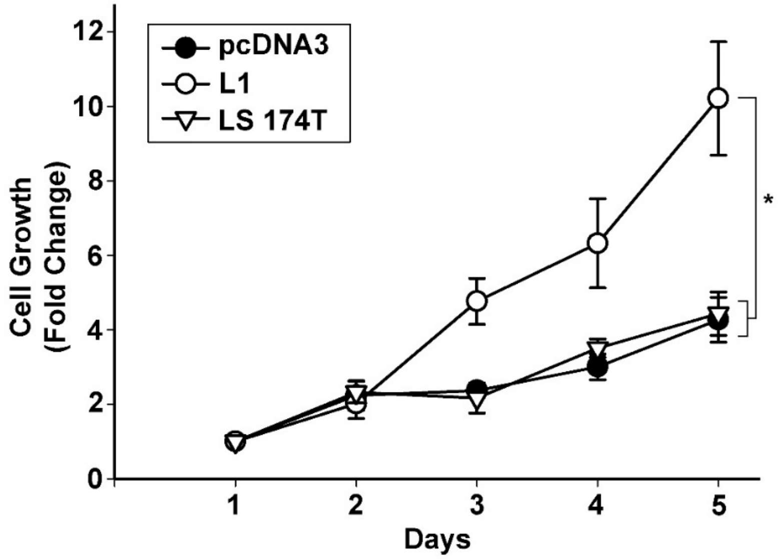

Supplementary Figure 1. The proliferation rate of untransfected LS 174T cells and of LS 174T cells stably transfected with the empty pcDNA3 plasmid and with the L1-containing pcDNA3 plasmid were compared for 5 days in the presence of 0.5% serum.

Table S2. Primers used for qRT-PCR experiment

**Gene**	**Forward**	**Reverse**
PLOD2	GACAGCGTTCTCTTCGTCCTCA	CTCCAGCCTTTTCGTGGTGACT
SMAD2	GGGTTTTGAAGCCGTCTATCAGC	CCAACCACTGTAGAGGTCCATTC
SMAD3	TGAGGCTGTCTACCAGTTGACC	GTGAGGACCTTGTCAAGCCACT
GAPDH	GTCTCCTCTGACTTCAACAGCG	ACCACCCTGTTGCTGTAGCCAA
